# Identification of Potential Risk Genes and the Immune Landscape of Idiopathic Pulmonary Arterial Hypertension via Microarray Gene Expression Dataset Reanalysis

**DOI:** 10.3390/genes12010125

**Published:** 2021-01-19

**Authors:** Jing Xu, Yicheng Yang, Yuejin Yang, Changming Xiong

**Affiliations:** 1 Department of Cardiology, State Key Laboratory of Cardiovascular Disease, Fuwai Hospital, National Center for Cardiovascular Diseases, Chinese Academy of Medical Sciences and Peking Union Medical College, Beijing 100037, China; Xujing0701@student.pumc.edu.cn; 2 Pulmonary Vascular Disease Center, State Key Laboratory of Cardiovascular Disease, Fuwai Hospital, National Center for Cardiovascular Diseases, Chinese Academy of Medical Sciences and Peking Union Medical College, Beijing 100037, China; yichengyang@student.pumc.edu.cn

**Keywords:** idiopathic pulmonary arterial hypertension, differentially expressed genes, immune cell infiltration, drug–gene interaction, bioinformatic

## Abstract

Gene dysfunction and immune cell infiltration play an essential role in the pathogenesis of idiopathic pulmonary arterial hypertension (IPAH). We aimed to investigate the immune landscape and novel differentially expressed genes (DEGs) of IPAH. In addition, potential druggable molecular targets for IPAH were also explored. In this study, the GSE117261 dataset was reanalyzed to explore the immune landscape and hub DEGs of IPAH. Lasso Cox regression analysis and receiver operating characteristic curve analysis were performed to detect the predictive value of IPAH. Additionally, the underlying drug targets for IPAH treatment were determined by drug–gene analysis. IPAH was significantly associated with the transforming growth factor-β (TGF-β) signaling pathway and Wnt signaling pathway as well as energetic metabolism dysfunction. We identified 31 upregulated and 39 downregulated DEGs in IPAH patients. Six hub genes, namely, *SAA1*, *CCL5*, *CXCR1*, *CXCR2*, *CCR1*, and *ADORA3*, were related to IPAH pathogenesis regardless of sex differences. Prediction model analysis showed that the area under the curve values of the hub DEGs except *CXCR2* were all above 0.9 for distinguishing IPAH patients. In addition, the relative proportions of 5 subtypes of immune cells, namely, CD_8_^+^ T cells, CD_4_^+^ memory resting T cells, γ delta T cells, M1 macrophages, and resting mast cells, were significantly upregulated in the IPAH samples, while 6 subtypes of immune cells, namely, CD_4_^+^ naive T cells, resting NK cells, monocytes, M0 macrophages, activated mast cells, and neutrophils, were downregulated. Additionally, a total of 17 intersecting drugs targeting 5 genes, *CCL5*, *CXCR1*, *CXCR2*, *CCR1*, and *ADORA3*, were generated as potential druggable molecular targets for IPAH. Our study revealed the underlying correlations between genes and immune cells in IPAH and demonstrated for the first time that *SAA1*, *CCL5*, *CXCR1*, *CCR1*, and *ADORA3* may be novel genetic targets for IPAH.

## 1. Introduction

Pulmonary arterial hypertension (PAH), defined as a mean pulmonary artery pressure ≥ 25 mmHg and pulmonary capillary wedge pressure ≤ 15 mmHg on resting right heart catheterization, is a progressive disease that may lead to right heart failure and hemodynamic disorder [1]. Despite the use of targeted drugs in the clinic, PAH remains a life-limiting disease. High pressure in the pulmonary artery is attributed to vasoconstriction, pulmonary vascular remodeling and vascular inflammation, and current research focuses on exploring more novel pathogenic mechanisms to reverse PAH; however, this research still far from clinical practice [2]. Several genetic targets and immune patterns of PAH have been revealed [3,4,5]. The gene spectrum and immune landscape have gained great attention for their value in reversing PAH.

PAH without a cause or associated condition is called idiopathic PAH (IPAH). Although genetic dysfunction is commonly regarded as the basic pathogenesis of IPAH, several known targeted genes explain only 15–30% of IPAH cases. Moreover, although recent studies have demonstrated that both bone morphogenetic protein (BMP) 9 [2] and prostacyclin synthase [6] genetic variants may also be involved in the pathogenesis of IPAH, they still fail to fully explain the cause of IPAH in patients, indicating that the genetic basis of IPAH needs further investigation. In addition, immune and inflammatory cells play essential roles in the pathology of IPAH [2], and studies of the immune landscape may be valuable for developing novel approaches to treat IPAH.

**Figure 1:**
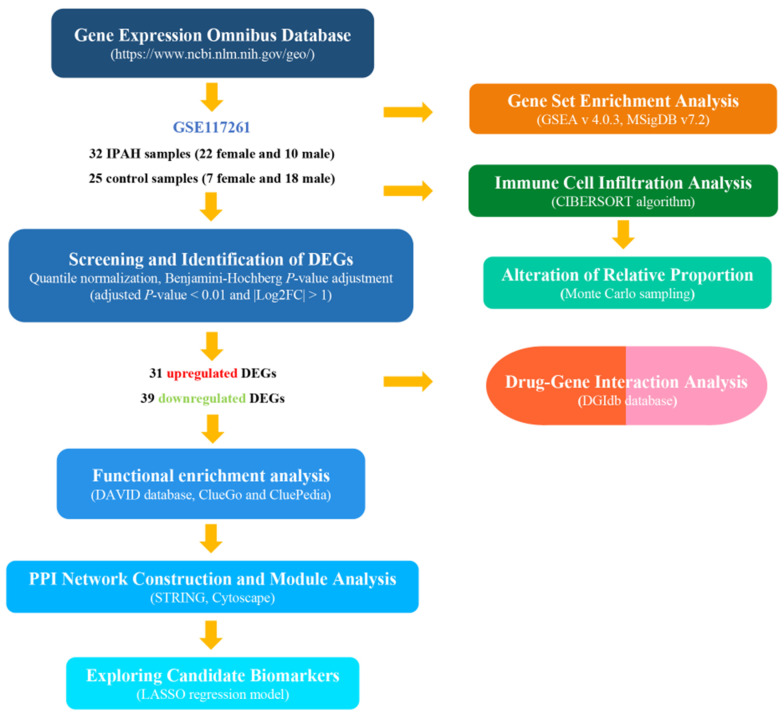
The overview of the analysis procedure. GSE117261 dataset profiles were downloaded from the Gene Expression Omnibus database, and Gene Set Enrichment Analysis (GSEA) was conducted to investigate the potential biological pathways using the entire gene set. Thirty-one common upregulated differentially expressed genes (DEGs) and 39 common downregulated DEGs were identified. The DAVID database, ClueGo and Clupedia were used to perform GO and pathway enrichment of the DEGs, and STRING was used to construct the PPI network. The hub genes were detected by Cytoscape software. The immune landscape in the dataset samples was determined by the CIBERSORT algorithm. Lasso Cox regression analysis and ROC analysis were performed to build the IPAH prediction model. Additionally, drug–gene analysis was conducted to explore underlying drug targets for IPAH treatment. GSEA: Gene set enrichment analysis; DEGs: Differentially expressed genes; GO: Gene ontology; PPI: Protein–protein interaction; ROC: Receiver operating characteristic.

Bioinformatic research has been used to investigate the potential pathogenic mechanisms of cardiovascular diseases [7]. In this study, the GSE117261 dataset profiles produced by Stearman et al. [8] were acquired from the Gene Expression Omnibus (GEO) database (https://www.ncbi.nlm.nih.gov/geo/). GSE117261 contains gene expression data from the complete transcriptomics analysis of IPAH and control lung biopsy tissues. To date, few data-based studies have been performed to analyze the potential genes and immune cell infiltration of IPAH. We analyzed the transcriptome differences and immune landscape of IPAH patients as well as potential druggable molecular targets for IPAH treatment, which may provide novel insights for disease development. Figure 1 shows the flowchart of the analysis procedure.

## 2. Materials and Methods

### 2.1. Data Resources

We downloaded the normalized gene expression profiles from the GEO database (https://www.ncbi.nlm.nih.gov/geo/) [9]. The GSE117261 dataset, tested on GPL6224 based on the Affymetrix Human Gene 1.0 ST Array included gene expression data from the complete transcriptomics analysis of PAH and control lung tissues. The dataset produced by Stearman et al. [8] contained 58 PAH and 25 control lung tissue samples. After excluding samples from patients diagnosed with other types of PAH, 32 IPAH samples and 25 normal control samples from failed donors were finally included in the subsequent analysis. To start, 33,297 gene probes were matched to the corresponding official gene symbol after the platform description matrix files were downloaded. After considering multiple probes that matched to one gene, retaining the probes with the most significant gene expression value (adjusted *p* value), and deleting the non-mRNA probes, 23,307 genes were identified. The following procedures were performed based on the matched matrix file.

### 2.2. Screening and Identification of Differentially Expressed Genes

We used the *limma* package to screen for differentially expressed genes (DEGs) between IPAH patients and healthy controls based on the R platform (R-project.org). The fold change (FC) value was obtained by calculating the ratio of the expression level of each gene between IPAH and control samples. Logarithmic operations with 2 as the base number were used to make easier comparisons. Genes with |log_2_ FC| ≥ 1 were considered DEGs, and to further limit the number of DEGs to facilitate the construction of the prediction model, an adjusted *p* value < 0.01 was considered the threshold value, corrected by the Benjamini–Hochberg method. DEGs with log_2_ FC < 0 were considered downregulated, whereas those with log_2_ FC > 0 were considered upregulated. The results were further validated by GEO2R, an online R-based web application supported by the GEO database [10].

### 2.3. Functional Analysis of the Expression Profiles

Gene Set Enrichment Analysis (GSEA) was performed to investigate the relevant biological pathways from an overall perspective using the original probe-matched matrix file of IPAH and normal control samples. GSEA software v4.0.3 was downloaded from the official website of the Broad Institute (http://www.broadinstitute.org/gsea) [11], and the analysis was conducted using the Molecular Signatures Database (MSigDB) of KEGG gene sets (c2.cp.kegg.v7.2.symbols). The normalized enrichment scores (NES) and nominal *p* values were generated by running GSEA. |NES| ≥ 1 and nominal *p* value < 0.05 were considered significant [12]. The GO enrichment analysis of the DEGs was performed by the ClueGO (version 2.5.7) and CluePedia (version 1.5.7) tool kits, which can decipher functionally grouped gene ontology (GO) and pathway annotation networks with a hypergeometric test and analyze functional correlations among pathways via Cytoscape software (version 3.7.1) [13,14,15]. To further validate and investigate the results of GO analysis, the biological process (BP), cellular component (CC), molecular function (MF), and KEGG pathway annotations of the hub genes were conducted via DAVID (http://david.ncifcrf.gov/, version 6.8). In particular, Homo sapiens was selected to limit the annotation of the species. A *p* value < 0.05 was considered the threshold value to explore more comprehensive GO results.

### 2.4. Protein Interaction and Module Analysis

The Search Tool for the Retrieval of Interacting Genes/Proteins (STRING, http://string-db.org/, version 11.0) was used to establish the protein–protein interaction (PPI) network of the DEGs [16]. The STRING database contains multisource information, including the integration of text mining in PubMed, experimental/biochemical evidence, coexpression, and database association to provide functional interactions between proteins. The DEGs were entered, and Homo sapiens was selected as the organism. To further narrow the candidate gene field, the highest confidence level of 0.90 was used. Then, the PPI network was constructed using Cytoscape software. The Molecular Complex Detection (MCODE, version 1.6.1) plug-in, a well-known automated kit based on topology to identify densely connected regions as molecular complexes in large PPI networks, was used to screen the modules of the PPI network. The MCODE parameter criteria were set by default as follows: Degree cutoff = 2, node score cutoff = 0.2, max depth = 100 and k-score = 2.

### 2.5. Evaluation of Immune Cell Infiltration

The normalized gene expression data with gene symbols were analyzed to infer the relative proportions of infiltrating immune cells of the selected samples via the CIBERSORT algorithm, a computational method for quantifying immune cell fractions from bulk tissue gene expression profiles based on gene expression reference values from a signature matrix of 547 genes in 22 types of immune cells. The modified expression file of GSE117261 was uploaded to the CIBERSORT website (http://cibersort.stanford.edu/), with the algorithm run by setting the default signature matrix at 1000 permutations. CIBERSORT generates a *p* value for the deconvolution for each sample using Monte Carlo sampling, offering a measure of confidence in the results. Significant alterations in immune cells between IPAH and control samples were identified according to the threshold of the Wilcoxon test at a *p* value < 0.05.

### 2.6. Prediction Model Analysis

The *glmnet* package in R software was utilized to calculate and select the linear models and preserve valuable variables by Lasso Cox regression analysis. According to the binary output variable in the processed data, we used a binomial distribution variable in the LASSO classification as well as the 1 standard error of the minimum criteria (the 1-SE criteria) lambda value in order to build the model with decent performance but the least number of variables. The expression level of the hub genes and the diagnosis of the 57 samples were obtained from the probe-matched matrix file. The drawing of the receiver operating characteristic (ROC) curves and the calculation of the area under the curve (AUC) were conducted by the *ROC* package in R, and the samples were randomly assigned to the training or testing cohort in an approximately 2:1 ratio. Thus, we investigated the feasibility of the hub genes in prediction via the AUC value.

### 2.7. Drug–Gene Interaction Analysis

The hub genes also served as potential targets in the search for drugs through the Drug–Gene Interaction database (DGIdb, http://www.dgidb.org/, version 3.0.2—sha1 ec916b2). This web-based database provides relevant drug–gene interaction data and gene druggability information from multiple sources, including clinical trial databases, web resources, and scientific papers in NCBI PubMed.

## 3. Results

### 3.1. Screening and Identification of Differentially Expressed Genes between IPAH and Control Samples

The available numerical expression values of 32 IPAH samples and 25 normal control samples from GSE117261 were used to identify DEGs. As shown in Table 1, compared with the control samples, there were 31 upregulated and 39 downregulated DEGs in the IPAH samples (|log_2_ FC| ≥ 1 and adjusted *p* value < 0.01) identified from the original transcript matrix (*n* = 33,297). The expression data with gene symbols are shown in Supplementary File 1.

### 3.2. Functional Annotation and Enrichment of the Expression Profiles

**Figure 2:**
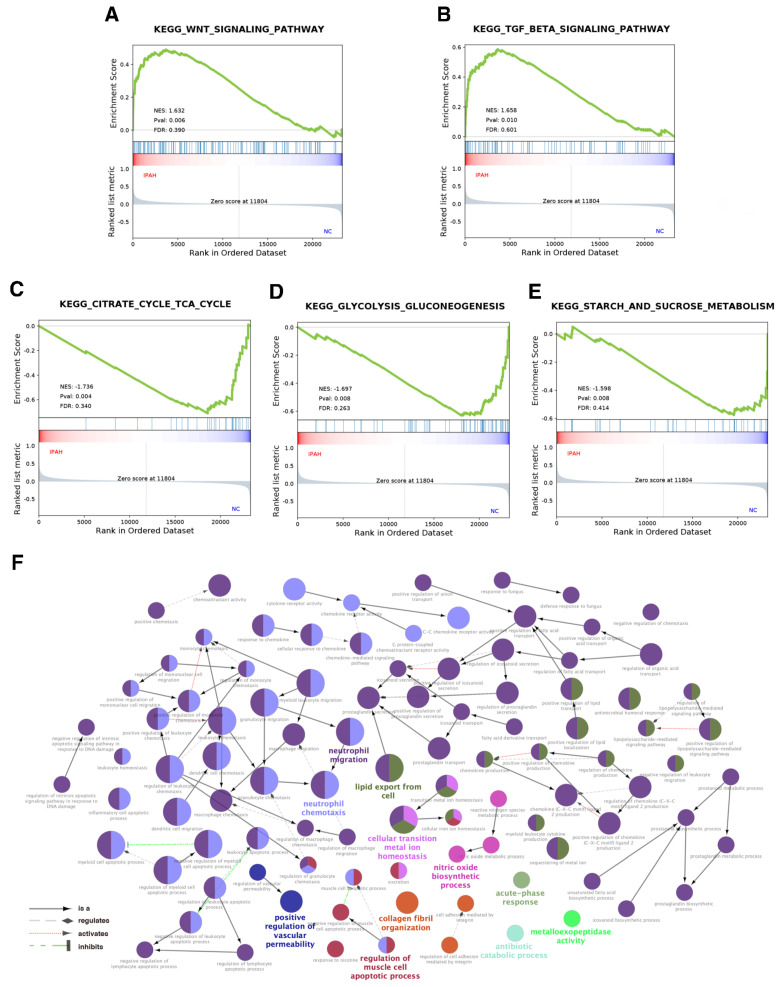
GSEA showed that (**A**) the Wnt signaling pathway and (**B**) TGF-β signaling pathway were positively associated with IPAH, while (**C**) the citrate cycle, tricarboxylic acid cycle, (**D**) glycolysis, gluconeogenesis, and (**E**) starch and sucrose metabolism were negatively associated with IPAH compared with normal control, all |NES| ≥ 1 and *p* value < 0.01. (**F**) The ontology relations between different GO terms. Different colors were used to distinguish the GO groups, and the bold font represents the leading group terms. GSEA: Gene set enrichment analysis; TGF–β: Transforming growth factor-β; GO: Gene ontology.

To explore the possible biological mechanisms to uncover the collective behavior of gene expression in states of IPAH and normal controls, GSEA was utilized to interpret the genes distributed across the entire network. IPAH was significantly associated with the transforming growth factor-β (TGF-β) and Wnt signaling pathways (Figure 2A,B, *p* value < 0.01) as well as relatively downregulated activities in energetic metabolism, including the citrate cycle, tricarboxylic acid cycle, glycolysis, gluconeogenesis, and starch and sucrose metabolism in IPAH samples compared with normal controls (Figure 2C,E, *p* value < 0.01). Additionally, we found that IPAH shared a number of KEGG pathways with cardiomyopathy, viral myocarditis, and melanogenesis, and details are shown in Figure S1, Figure S2, and Supplementary File 2. GO analysis of the DEGs was conducted via the ClueGO and CluePedia tool kits in Cytoscape. As shown in Table 2, a total of 149 significant GO terms (*p* value < 0.01, see Supplementary File 3 for details) were classified into 11 groups according to Cohen’s kappa score based on the shared genes between the terms [11]. The ontology relations between different GO terms are shown in Figure 2F.

### 3.3. Evaluation of Immune Cell Infiltration

**Figure 3:**
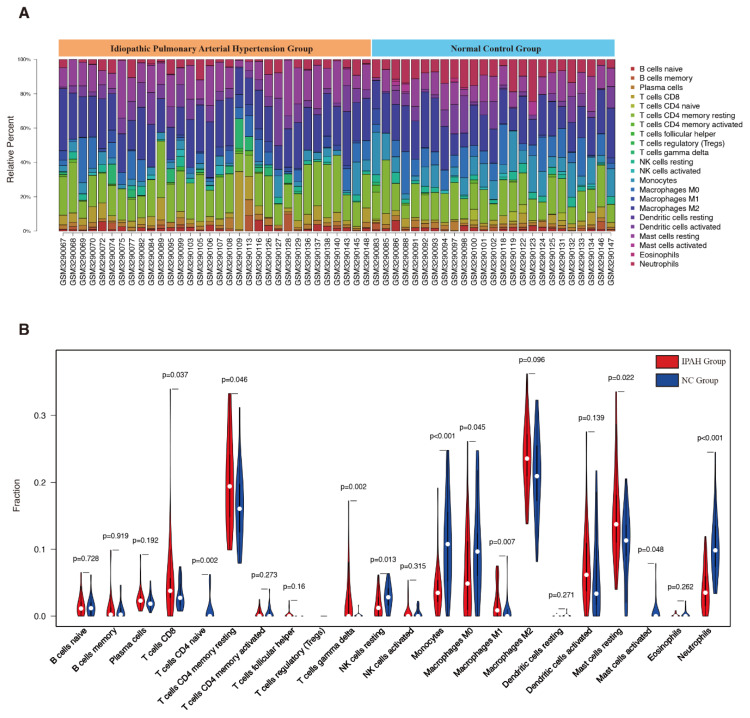
(**A**) Results of immune landscape analysis. (**B**) Different immune cell infiltration between IPAH and control samples. NC: Normal control.

The CIBERSORT algorithm was used to investigate the infiltration percentages of 22 subpopulations of immune cells in the IPAH and control samples from GSE117261. The relative percentage of each cell in 32 IPAH and 25 control samples is shown in Figure 3A. Moreover, as shown in Figure 3B, the relative proportions of 11 subtypes of immune cell were significantly different and objectively detectable between IPAH and control samples. CD_8_^+^ T cells (*p* = 0.037), CD_4_^+^ memory resting T cells (*p* = 0.046), γ delta T cells (*p* = 0.002), M1 macrophages (*p* = 0.007), and resting mast cells (*p* = 0.022) were upregulated in IPAH samples, while CD_4_^+^ naive T cells (*p* = 0.002), resting NK cells (*p* = 0.013), monocytes (*p* < 0.001), M0 macrophages (*p* = 0.045), activated mast cells (*p* = 0.048), and neutrophils (*p* < 0.001) were downregulated in IPAH samples. The heatmap of CIBERSORT analysis and measures of confidence are shown in Supplementary File 4.

### 3.4. Protein Interaction and Module Analysis

**Figure 4:**
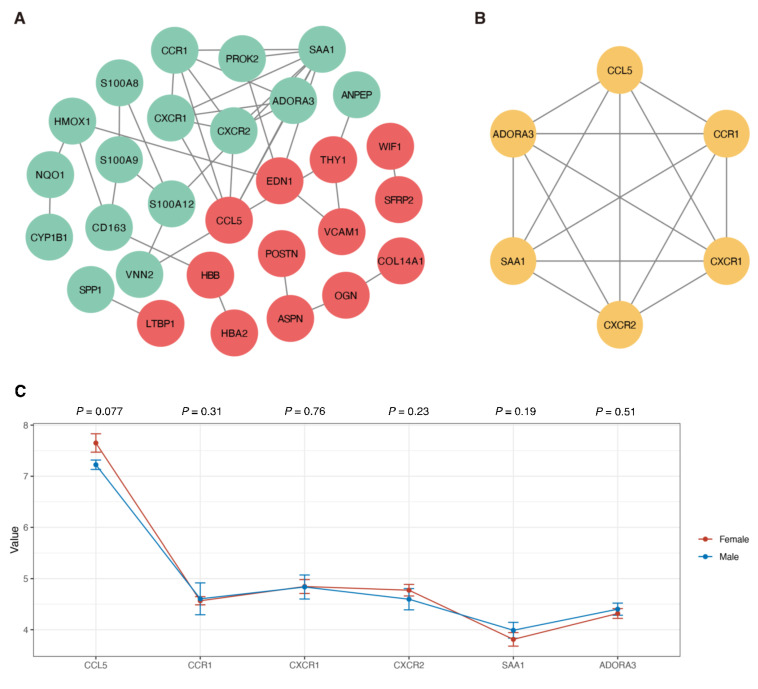
(**A**) PPI network analysis of DEGs. The red circles indicate the upregulated genes, while the green circles represent the downregulated genes. (**B**) The modules with the highest scores in the PPI network. The yellow circles represent the hub genes. (**C**) The expression level of the hub genes in the biopsy sample from female and male patients. The Wilcoxon rank-sum test was conducted on the male and female patients according to each hub gene, and the *p* value is displayed at the top of the figure. PPI: Protein–protein interaction; DEGs: Differentially expressed genes.

To construct the PPI network of DEGs, the STRING online database and Cytoscape software were utilized. A total of 70 DEGs were filtered into the PPI network, which included 29 nodes and 39 edges (Figure 4A). Based on the confidence level of 0.90, 41 genes were not included in the PPI network. According to the node degree >c5 criterion, the 6 hub genes were *SAA1* (degree = 8), *CCL5* (degree = 5), *CCR1* (degree = 5), *CXCR2* (degree = 5), *CXCR1* (degree = 5), and *ADORA3* (degree = 5). The MCODE plug-in was used to analyze the significant modules, and a module with 6 nodes with 15 edges was selected from the PPI network (Figure 4B), showing that the results were consistent with the 6 hub genes. We also conducted the Wilcoxon rank-sum test to investigate the expression values of these hub genes in different samples based on sex, and as shown in Figure 4C, the expression of *CCL5* (*p* = 0.077), *CCR1* (*p* = 0.31), *CXCR1* (*p* = 0.76), *CXCR2* (*p* = 0.23), *SAA1* (*p* = 0.19), and *ADORA3* (*p* = 0.51) was not significantly different between males and females. The significant functional annotations, including BP, CC, MF, and KEGG pathways, are shown in Table 3.

### 3.5. Exploring Candidate Biomarkers by Lasso Regression and Receiver Operating Characteristic Curves

**Figure 5:**
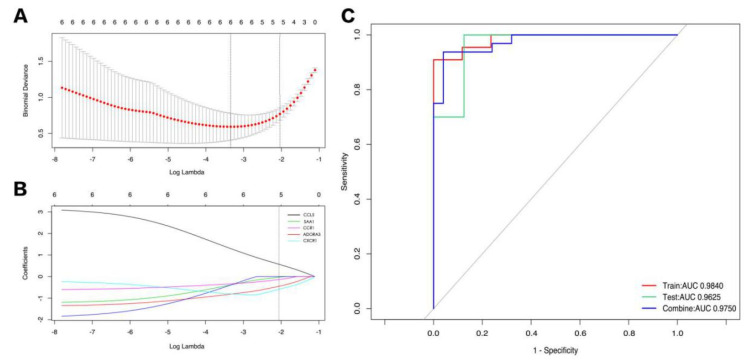
(**A**,**B**) Lasso regression model for the hub DEGs of IPAH and control samples from GSE117261. (**C**) Results of ROC curve analysis of the Lasso regression model. DEGs: Differentially expressed genes; ROC: Receiver operating characteristic.

First, a Lasso regression model for the hub DEGs of IPAH and control samples from GSE117261 was established to determine an optimum linear combination for predicting IPAH (Figure 5A,B), with coefficients of −0.5826, 0.5619, −0.4437, −0.1321, and −0.028 for *CXCR1*, *CCL5*, *ADORA3*, *CCR1*, and *SAA1*, respectively. Then, ROC curve analysis of the Lasso regression model was conducted separately to predict IPAH in the training cohort, testing cohort, and combination cohort, and the AUC values were all above 0.9, which suggests that the genes in the model might have outstanding potential to as biomarkers for distinguishing IPAH patients (Figure 5C).

### 3.6. Drug–Gene Interaction Analysis

The drug–gene interaction network of the hub genes was screened via the DGIdb database (http://www.dgidb.org/), aiming to identify druggable targets. As shown in Table 4, a total of 17 intersecting drugs targeting 5 genes, *CCL5*, *CXCR1*, *CXCR2*, *CCR1*, and *ADORA3*, were generated as potential druggable molecular targets for IPAH.

## 4. Discussion

IPAH, a rare but life-threatening disease, remains challenging in terms of its diagnosis and treatment, leading to a 5-year survival rate of approximately 50% [17,18] even with the administration of targeted drugs. Investigations of effective treatment strategies and the underlying methods are still needed. Genetic dysfunction is commonly recognized as the underlying pathogenesis of IPAH, and immune disorders also play an essential role in disease progression [2]. Recently, the exploration of gene dysfunction and the immune landscape in IPAH has received unprecedented attention due to their great potential for reconstructing therapeutic ideas, which may improve the unsatisfactory treatment situation of IPAH. Stearman et al. [8] conducted the largest PAH lung transcriptome study to date to provide insights into therapies and generate novel hypotheses for preclinical testing. Their study included patients diagnosed with group 1 PH, including IPAH, associated PAH, heritable PAH, and others, for analysis. Here, we specifically focused on the transcriptome differences and immune landscape between IPAH and control samples.

Our study demonstrated that IPAH was significantly associated with upregulation of both the TGF-β signaling pathway and Wnt signaling pathway. The TGF-β signaling pathway, closely related to inflammation, plays a vital role in numerous biological processes by regulating cell growth, differentiation, apoptosis, and cellular homeostasis, etc., and dysfunction is associated with the occurrence of cancer, immune disease as well as cardiovascular diseases [19,20]. An increasing amount of evidence has demonstrated the essential role of inflammation in the pathogenesis of IPAH [21,22], and the underlying mechanisms between the TGF-β signaling pathway and IPAH are currently under heated exploration. TGF-β/activin/nodal signaling, one of the TGF-β signaling pathways, branches through Smad2/3. After pSmad2/3 oligomerizes with Smad 4, they translocate into the nucleus to regulate the transcription of target genes, exerting effects on pulmonary vascular remodeling and pulmonary artery smooth muscle cell proliferation. In addition, dysregulated TGF-β/activin/nodal signaling enables the activation of extracellular-signal-regulated kinase, nuclear factor-κB and Rho kinase pathways, which may also promote PAH [23]. A clinical study [24] noted that a higher level of TGF-β1 could be identified in patients with IPAH compared with the control group. The Wnt signaling pathway is of utmost importance in regulating proliferation and differentiation [25]. Upregulation of the Wnt signaling pathway is regarded as the pathogenesis of both IPAH and heritable PAH [26]. Meanwhile, Hemnes et al. [27] also determined that higher increased stimulated Wnt signaling pathway activity in IPAH patients than in the control group could be detected after analyzing human lung fibroblasts. Moreover, dysfunction of energetic metabolism in terms of downregulation of the tricarboxylic acid cycle in IPAH has also been demonstrated, which uncovers IPAH as an energy metabolism-related disease.

The immune landscape provides a deeper understanding of the inflammatory components in the pathogenesis of IPAH, which helps in the investigation of novel treatments. Our results showed that CD_8_^+^ T cells, CD_4_^+^ memory resting T cells, γ delta T cells, M1 macrophages, and resting mast cells were upregulated, while CD_4_^+^ naive T cells, resting NK cells, monocytes, M0 macrophages, activated mast cells, and neutrophils were downregulated in IPAH samples. It has been reported that varying degrees of perivascular inflammatory infiltrates, such as T- and B-lymphocytes, mast cells, macrophages, and dendritic cells, occur in PAH patients or animal models [28]. Similar to our findings, Marsh et al. [22] also showed that CD_4_^+^, CD_8_^+^, and γ delta T-cell subsets were also increased in the lungs of patients with IPAH. CD_4_^+^ and CD_8_^+^ T cells are able to induce proinflammatory cytokine release, leading to pulmonary artery injury. γ delta T-cells, serving as a link between innate and adaptive immune responses, help tissue homeostasis and wound healing by releasing insulin-like growth factor-1, which exerts a proliferative promotion effect on smooth muscle cells to induce IPAH [9,29]. M1 macrophages, also called classically activated macrophages, produce proinflammatory cytokines such as IL-1β, TNF, IL-12, and IL-18 [30], and increased inflammatory markers can exacerbate damage to pulmonary vessels. The study showed that inflammation and vascular smooth muscle cell phenotypic switching induced by activated M1 macrophages are related to the increased expression of carbonic anhydrase 2. The use of carbonic anhydrase inhibitors exerts an immunomodulatory effect to treat macrophage-mediated inflammation [31]. Mast cells are the first immune cells recognized in pulmonary vascular lesions in IPAH patients and can release cysteinyl leukotriene C4 and endothelin to enhance lung vascular remodeling and PH pathogenesis [32]. Our results showed that resting mast cells, not activated mast cells, were increased in IPAH patients. Similarly, Wang et al. [33] revealed that resting mast cells were increased in idiopathic pulmonary fibrosis, which has been regarded as another immune disorder disease, and the role of resting mast cells in the pathogenesis of lung immune diseases such as IPAH may be worthy of exploration. Recently, reductions in NK cells in both PAH mouse models and PAH patients were identified. The dysfunction of NK cells has been regarded as an important regulator of angiogenesis and vascular remodeling, potentially according to the induction of angiogenic factors and chemokines [34,35]. A clinical study [36] showed that after 1 year of follow-up, PAH patients, including IPAH patients with deficiencies in NK cells and cytotoxic CD_8_^+^ T cells, were deceased, while patients with normal lymphocyte profiles were all alive. Decreased NK cells are linked to a high risk of death, but the underlying mechanisms are still unclear. This suggests that NK cell depletion may be a consequence of or a predisposing factor for PAH, and the association between programmed death-1 expression on NK cells and disease progression needs further investigation [35]. Monocytes induce cytokines to promote inflammation and remodeling [37,38]. Neutrophils release proteolytic enzymes that modulate the activity of cytokines and degrade the extracellular matrix, releasing growth factors to promote vascular remodeling. Moreover, proteolytic enzymes may also alter the inflammatory environment, enhance leukocyte responses, and exacerbate inflammatory effects [39,40]. It seems that monocyte and neutrophil infiltration contributes to the pathogenesis of PAH, while our study revealed relatively lower fractions of these cells in IPAH samples. PAH-targeted drugs can inhibit inflammatory effects [41,42], and the reduction in inflammatory cells, including monocytes and neutrophils, may be attributed to the use of PAH drugs in our samples. Novel insights into the promising correlations and mechanisms are needed. Research on the immune landscape provides prospective evidence of IPAH pathogenesis, which can be used to explore more treatment strategies via further investigation.

In our study, a total of 70 DEGs were identified. Similar to Stearman’s study [8], we found that upregulated genes such as *CCL5*, *VCAM1*, and *EDN1* and downregulated genes including *CXCR2* were also identified in IPAH. We first demonstrated that the dysregulation of *SAA1* (log_2_ FC = −1.57), *CCR1* (log_2_ FC = −1.04), *CXCR1* (log_2_ FC = −1.24), and *ADORA3* (log_2_ FC = −1.03) may also play essential roles in the pathogenesis of IPAH. After reanalyzing the GSE117261 series matrix dataset, 6 hub DEGs of IPAH in terms of *SAA1* (degree = 8), *CCL5* (degree = 5), *CCR1* (degree = 5), *CXCR2* (degree = 5), *CXCR1* (degree = 5), and *ADORA3* (degree = 5) were identified in our study, and no significance was found in the comparison of female and male patients, indicating that these hub DEGs were not a result of sex-associated discrepancies. *SAA1*, located on the short arm of chromosome 11, showed the highest degree of connectivity with IPAH. The SAA protein, encoded by *SAA1*, is highly induced during the acute-phase response and plays an important role in lipid metabolism, bacterial clearance, and tumor pathogenesis [43,44]. Recently, the function of the SAA protein in regulating inflammation has been fully discussed, and studies have consistently demonstrated that SAA [45,46] induces the expression of proinflammatory factors. One study suggested recombinant human SAA as an inflammatory cytokine, but it does not belong to any family of known chemokines and inflammatory cytokines due to its different structure [47]. In addition, anti-inflammatory factors such as IL-1R antagonist and IL-10 can also be induced by recombinant human SAA, suggesting that the primary role of SAA during inflammation may be homeostatic [48,49,50]. Our study showed that the downregulation of the *SAA1* gene was related to IPAH and hypothesized that the underlying mechanism may be attributed to the dysfunction of inflammation homeostasis. However, ongoing and future studies are warranted to explore powerful evidence on the association between SAA1 and IPAH. Other potential gene targets of IPAH, including *CCL5*, *CCR1*, *CXCR2*, *CXCR1*, and *ADORA3*, are all associated with inflammatory-immune regulation. IPAH is a kind of immune-mediated inflammatory disease [28]. Similar to previous studies, we revealed that the upregulated *CCL5* gene was a risk factor for the pathogenesis of PAH [51,52,53]. *CCL5* is one of the members of the CC-chemokine family, having a complex impact on immune cells such as monocytes, T lymphocytes, and NK cells [54]; it is strongly expressed on vascular endothelial cells and exerts vasoconstriction and remodeling effects on the lung tissue of patients with PAH [52,55]. Interestingly, *CCL5* interacts with bone morphogenetic protein receptor type-2 (*BMPR2*), which is regarded as an identified IPAH pathogenic gene [56,57]. Nie et al. [58] showed that PAH patients with decreased BMPR2 expression have higher expression levels of *CCL5* in pulmonary artery endothelial cells. The deletion of *CCL5* inhibited pulmonary vascular remodeling in mice by restoring *BMPR2* and activating the phosphorylation of BMP target proteins. In addition, the reduction in *CCL5* improved pulmonary artery endothelial cell survival and suppressed the proliferation of pulmonary artery smooth muscle cells to reverse IPAH through BMPR2 signal enhancement. The adenosine A3 receptor (A3R) coupled to Gi proteins is encoded by *ADORA3*, suggesting that it is a regulator of inflammatory responses [59]. The role of this receptor in the pathophysiology of inflammation is complex. Putten et al. [60] pointed out that A3R-mediated signaling induced proinflammatory cytokines, while another study showed the protective effect of preventing excessive immune response and immune-mediated damage after A3R activation [61]. To date, research on downregulated *ADORA3* and IPAH pathogenesis is insufficient, and the underlying mechanisms still need to be explored.

More importantly, the ROC curve analysis of the Lasso regression model showed that the AUC values were all above 0.9, indicating the outstanding potential of the 5 hub DEGs, namely, *CXCR1*, *CCL5*, *ADORA3*, *CCR1*, and *SAA1*, as biomarkers for distinguishing IPAH patients, which has significant clinical feasibility in auxiliary diagnosis and disease classification. In Stearman’s study [8], differentially regulated drug targets in PAH, including *EDN1*, *EDNRA*, *PDE5A*, *GUCY1B1*, *PTGIR*, *PTGIS*, and *CACNA1C*, which are related to the endothelin pathway, phosphodiesterase family, prostanoid pathway proteins, and voltage-gated calcium channels, were demonstrated. However, in this study, we showed that *CCL5*, *CXCR1*, *CXCR2*, *CCR1*, and *ADORA3*, associated mainly with inflammatory and immune pathways, were all identified as potential druggable molecular targets for IPAH, which might also reflect the inflammatory and immune pathogenesis of IPAH. Currently, IPAH pharmacotherapy, except for classic targeted drugs related to the nitric oxide pathway, endothelin pathway, and prostacyclin pathway, is still limited. The development of a treatment strategy relies on full insights into the pathogenesis of IPAH. Here, we highlight that the dysfunctions of 6 genes with great potential induce IPAH, and investigational drugs targeting *CCL5*, *CXCR1*, *CXCR2*, *CCR1*, and *ADORA3* may provide a prospective direction for the treatment of IPAH. Our work is promising with regards to advancements in treating this devastating condition, which may significantly the prognosis of patients.

However, several limitations remain in our study. First, our results were based on GSE117261, and other datasets or clinical data are needed for further research. In this study, all of the samples were collected from Caucasians, which may cause selection bias. Second, smoking exposure could alter the inflammatory status of the lungs. SAA is also reported to be an indicator of the inflammatory status of the lung associated with an increased risk of developing lung cancer in heavy smokers. However, in this dataset, no smoking or cancer data are provided. Third, biomarkers exploration in our study has not been verified by external IPAH cohorts and in the future other cohorts are eager to conduct to test candidate biomarkers mentioned in our study. Also, biological experiments are needed for further verification.

## 5. Conclusions

In conclusion, therapeutic strategies for IPAH are currently limited due to the complex pathogenesis of IPAH. The dysregulation of genes and immune cell infiltration are regarded as important mechanisms that promote disease progression. Our study demonstrated related signaling pathways and the immune landscape of IPAH as well as identified 6 hub genes, which might help to further provide novel insights for candidate biomarker exploration and treatment development in IPAH.

## Figures and Tables

**Table 1:** The common differentially expressed genes from GSE117261.

	DEGs
Upregulated DEGs	*HBB, LTBP1, HBA2, PDE3A, CCL5, BMP6, MFAP4, ABCG2, RGS5, WIF1, SFRP2, EDN1, ASPN, COL14A1, OGN, DPT, RGS1, CD69, C10orf10, ESM1, GZMK, MXRA5, AGBL1, ENPP2, POSTN, VCAM1, CPA3, FABP4, IFI44L, ROBO2, THY1*
Downregulated DEGs	*CSF3R, RNASE2, S100A9, MGAM, AQP9, SULT1B1, CR1, S100A8, NQO1, S100A12, LILRB3, CXCR1, IL1R2, NPL, PROK2, CXCR2, ADORA3, CD163, SIGLEC10, ANPEP, HMOX1, CCR1, VNN2, SAA1, LCN2, ELF5, GPR110, SPP1, SLC16A6, BPIFA1, CTSE, TIMP4, BPIFB1, SLC7A11, PLA2G7, CYP1B1, MT-TW, SERPINA3, SLCO4A1*

DEGs were set as adjusted *p* value < 0.01 and |log_2_ FC| ≥ 1. DEGs: Differentially expressed genes.

**Table 2:** The leading 11 GO terms.

GOID	GO Term	*p* Value	Percentage of Term
GO:1990266	Neutrophil migration	1.61 × 10^−9^	52.35%
GO:0030593	Neutrophil chemotaxis	3.91 × 10^−10^	22.15%
GO:0140353	Lipid export from cell	1.08 × 10^−6^	10.74%
GO:0010660	Regulation of muscle cell apoptotic process	1.35 × 10^−4^	4.7%
GO:0046916	Cellular transition metal ion homeostasis	5.74 × 10^−6^	2.68%
GO:0030199	Collagen fibril organization	4.34 × 10^−5^	2.01%
GO:0006809	Nitric oxide biosynthetic process	2.29 × 10^−4^	2.01%
GO:0043117	Positive regulation of vascular permeability	2.93 × 10^−5^	1.34%
GO:0017001	Antibiotic catabolic process	2.67 × 10^−3^	0.67%
GO:0008235	Metalloexopeptidase activity	2.18 × 10^−3^	0.67%
GO:0006953	Acute-phase response	1.74 × 10^−3^	0.67%

**Table 3:** Signaling pathway enrichment analysis of the significant functional annotations related to the common differentially expressed genes.

Category	Term	Count	*p*-Value	Genes
GOTERM_BP_DIRECT	GO:0006935: Chemotaxis	3	1.38 × 10^−4^	*CCR1, CXCR1, CXCR2*
GOTERM_BP_DIRECT	GO:0090026: Positive regulation of monocyte chemotaxis	2	6.32 × 10^−3^	*CCR1, CCL5*
GOTERM_BP_DIRECT	GO:0006953: Acute-phase response	1	6.32 × 10^−3^	*SAA1*
GOTERM_BP_DIRECT	GO:0070098: Chemokine-mediated signaling pathway	2	1.789 × 10^−2^	*CXCR2, CCL5*
GOTERM_BP_DIRECT	GO:0060326: Cell chemotaxis	1	1.98 × 10^−2^	*SAA1*
GOTERM_BP_DIRECT	GO:0034364: High-density lipoprotein particle	1	5.59 × 10^−3^	*SAA1*
GOTERM_BP_DIRECT	GO:0005615: Extracellular space	2	4.37 × 10^−2^	*SAA1, CCL5*
GOTERM_BP_DIRECT	GO:0042056: Chemoattractant activity	2	4.65 × 10^−5^	*SAA1, CCL5*
GOTERM_BP_DIRECT	GO:0016494: C-X-C Chemokine receptor activity	2	3.61 × 10^−3^	*CXCR1, CXCR2*
KEGG_PATHWAY	cfa04062: Chemokine signaling pathway	4	6.06 × 10^−5^	*CCR1, CXCR1, CXCR2, CCL5*
KEGG_PATHWAY	cfa04060: Cytokine–cytokine receptor interaction	4	9.44 × 10^−5^	*CCR1, CXCR1, CXCR2, CCL5*

BP: Biological process.

**Table 4:** Potential druggable molecular targets for idiopathic pulmonary arterial hypertension (IPAH).

Gene	Drug	Interaction Type
*CCL5*	FLUTICASONE PROPIONATE	anti-inflammatory agent
*CXCR1*	CHEMBL411250	agonist
*CXCR1*	PROPOFOL	agonist
*CXCR1*	CHOLINE ALFOSCERATE	agonist
*CXCR2*	PROPOFOL	agonist
*CXCR2*	BENZPIPERYLON	agonist
*CXCR2*	CHEMBL411250	agonist
*CXCR2*	MEPHENTERMINE	agonist
*CCR1*	ENOXAPARIN	agonist
*CCR1*	GLYCERIN	agonist
*CCR1*	GUANIDINO ACETATE	agonist
*CCR1*	GUANINE	agonist
*ADORA3*	IB-MECA	agonist
*ADORA3*	ADENOSINE	agonist
*ADORA3*	CF102	agonist
*ADORA3*	CHEMBL175543	agonist
*ADORA3*	CHEMBL472925	agonist

## Data Availability

No new data were created or analyzed in this study. Data sharing is not applicable to this article.

## Supplementary Materials

The following are available online at https://www.mdpi.com/2073-4425/12/1/125/s1, Figure S1: Other GSEA results of IPAH, Figure S2: Other GSEA results of control group, File 1: Seventy DEGs detailed information, File 2: Details of KEGG results, File 3: Detailed information of 149 significant GO terms. File 4: The heatmap of CIBERSORT analysis and measures of confidence.

## Abbreviations

IPAH	idiopathic pulmonary arterial hypertension;
PAH	pulmonary arterial hypertension;
DEGs	differentially expressed genes;
FC	fold change;
GO	gene ontology;
GEO	gene expression omnibus;
PPI	protein–protein interaction;
GSEA	gene set enrichment analysis;
MSigDB	molecular signatures database;
NES	normalized enrichment scores;
STRING	search tool for the retrieval of interacting genes/proteins;
MCODE	molecular complex detection;
DGIdb	drug–gene interaction database;
ROC	receiver operating characteristic;
AUC	area under the curve;
BP	biological process;
CC	cellular component;
MF	molecular function;
BMP	bone morphogenetic protein;
TGF-β	transforming growth factor-β.
